# The Impact of Information Presentation and Cognitive Dissonance on Processing Systematic Review Summaries: A Randomized Controlled Trial on Bicycle Helmet Legislation

**DOI:** 10.3390/ijerph19106234

**Published:** 2022-05-20

**Authors:** Benoît Béchard, Joachim Kimmerle, Justin Lawarée, Pierre-Oliver Bédard, Sharon E. Straus, Mathieu Ouimet

**Affiliations:** 1 PolitiCo, School of Psychology, Université Laval, Québec, QC G1V 0A6, Canada; benoit.bechard.1@ulaval.ca; 2 Leibniz-Institut für Wissensmedien, 72076 Tübingen, Germany; 3 International Observatory on the Societal Impact of AI and Digital Technology, Department of Political Science, Université Laval, Québec, QC G1V 0A6, Canada; justin.lawaree@observatoire-ia.ulaval.ca; 4 GC Experimentation Team, Treasury Board of Canada Secretariat, Government of Canada, Ottawa, ON K1A OR5, Canada; pierre-olivier.bedard.2@ulaval.ca; 5 Knowledge Translation Program, St. Michael’s Hospital, Toronto, ON M5B 1W8, Canada; sharon.straus@unityhealth.to; 6 PolitiCo, Department of Political Science, Université Laval, Québec, QC G1V 0A6, Canada; mathieu.ouimet@pol.ulaval.ca

**Keywords:** cognitive dissonance, systematic review summary, bicycle helmet, methodological limitations, experiments

## Abstract

*Background*: Summaries of systematic reviews are a reference method for the dissemination of research evidence on the effectiveness of public health interventions beyond the scientific community. Motivated reasoning and cognitive dissonance may interfere with readers’ ability to process the information included in such summaries. *Methods*: We conducted a web experiment on a panel of university-educated North Americans (*N* = 259) using a systematic review of the effectiveness of bicycle helmet legislation as a test case. The outcome variables were the perceived tentativeness of review findings and attitude toward bicycle helmet legislation. We manipulated two types of uncertainty: (i) deficient uncertainty (inclusion vs. non-inclusion of information on limitations of the studies included in the review) and (ii) consensus uncertainty (consensual findings showing legislation effectiveness vs. no evidence of effectiveness). We also examined whether reported expertise in helmet legislation and the frequency of wearing a helmet while cycling interact with the experimental factors. *Results*: None of the experimental manipulations had a main effect on the perceived tentativeness. The presentation of consensual efficacy findings had a positive main effect on the attitude toward the legislation. Self-reported expertise had a significant main effect on the perceived tentativeness, and exposing participants with reported expertise to results showing a lack of evidence of efficacy increased their favorable attitude toward the legislation. Participants’ helmet use was positively associated with their attitude toward the legislation (but not with perceived tentativeness). Helmet use did not interact with the experimental manipulations. *Conclusions*: Motivated reasoning and cognitive dissonance influence a reader’s ability to process information contained in a systematic review summary.

## 1. Introduction

### 1.1. Background

In comparison to other types of literature reviews, systematic reviews provide a more comprehensive and reliable source of evidence [1]. They cover all available research studies that address a question, and they do so through systematic procedures to identify, select and evaluate research [2]. In addition, they extract and analyze data from the studies that meet explicit inclusion criteria. Systematic reviews, which are part of the methodological arsenal of meta-research [3,4], provide an analysis of the limitations or uncertainty of relevant studies by drawing on a growing library of methodological tools [5,6]. This type of synthesis frequently results in long reports not always tailored to the needs of the users [7,8]. Researchers are pondering the most effective methods of summarizing the content of systematic reviews [8,9,10,11,12,13,14,15,16,17,18,19]. On the other hand, evidence summaries were found to have a negligible effect on readers’ knowledge and understanding [20], despite the fact that they are easier to comprehend than full-length systematic reviews. One proposed solution to this issue is to develop summary formats in close collaboration with their intended users [19,20].

These efforts to develop evidence summaries are commendable, since they aim to increase the use of research evidence while considering deficient uncertainty documented in the evaluation of study limitations. These systematic review summaries fit perfectly with the evidence-based decision-making movement [21], which encourages people to follow the “best” available research evidence in forming their opinions. This movement argues for these summaries to take precedence over other approaches to opinion formation, such as tenacity, authority, or a priori reasoning (on these alternative approaches, see Peirce’s classic work [22]). These summaries could then promote the social acceptability of public health interventions that are sometimes coercive, especially when they tell people without symptoms how to stay healthy, when it is based on the idea that the intervention does more good than harm, or when the people who criticize it are singled out for not following the recommendations [23]. However, human cognition is limited when performing complex tasks, such as comprehending text or challenging higher-order cognitive processes [24]. Hence, motivated reasoning (i.e., the tendency to match the evaluation of information to a personal goal extrinsic to the accuracy of such information [25]) may interfere with readers’ ability to process information. Very little is known about whether readers effectively process the information on the limitations of studies included in a systematic review summary. Do readers pay attention to this information? Does motivated reasoning diminish the influence that such information should ideally have on readers’ decision to consider the review findings as more or less tentative? Does reporting information on limitations influence readers’ attitudes toward the intervention assessed in the review? Does a summary that reports consensual findings favoring an intervention influence readers’ attitudes toward the intervention? These questions are essential, as they address the ability of systematic review summaries to foster the development of informed critical thinking among readers, which is at the heart of the foundations of evidence-based decision-making.

In the following sub-sections, we introduce the research hypotheses in the context of the literature. Subsequently, we present the materials and methods, followed by the experimental results. We conclude by discussing the implications of the findings for science communication through the dissemination of systematic summaries.

### 1.2. Research Hypotheses

The science literacy deficit has long been the preferred explanation for the lack of public understanding of science [26]. Faced with the mixed results that improving scientific literacy has on the public understanding of science [27], many researchers are now focusing on how to communicate science [28], an approach that is notably taken by developers of systematic review summaries. With a vision of a rational readership, these developers expect that the summarized information about study limitations and findings will be unambiguously understood and processed by the readers, especially when the summary uses a format that was carefully designed based on rigorously collected feedback from potential users (see [19]). The idea is that rational readers are guided by their motivation to assimilate information to maximize learning and not by subjective beliefs that would interfere with their understanding of the content of the summary. Following this rational perspective, processing uncertain information demonstrating that the studies synthesized in a systematic review have methodological limitations should increase the readers’ perceived tentativeness of the review findings. Consistent with this rational perspective, experimental studies exposed university students to media articles reporting the results of scientific research on the effectiveness of a specific health technology [29,30]. The results show that increasing the amount of verbal information on the study’s limitations had a main positive effect on the readers’ perceived tentativeness of research findings [29,30]. An internal meta-analysis of five experiments found that providing explicit verbal uncertainty decreased participants’ perceived reliability of the reported scientific numbers [31].

On the other hand, a cognitive dissonance view of information processing would deem reading comprehension a complex task, which involves establishing relationships among different parts of the text (and between the text) and the reader’s memories, knowledge, and experience (see [32]). In such cases, recent evidence shows that information tends to be perceived as reliable and informative if it is consistent with an individual’s prior beliefs and attitudes [33,34]. This confirmation bias mechanism is drawn from the cognitive dissonance theory under the belief disconfirmation paradigm (see the seminal work by [35]; see also [36]). Inconsistency between prior beliefs (cognition 1) and information in the summary of a systematic review (cognition 2) can cause readers to reject the information, thus allowing them to escape dissonant information. According to this cognitive dissonance perspective, one could expect that disclosing information on limitations in a systematic review summary will not necessarily influence readers’ perceived tentativeness of the review findings. Some studies have found that reporting limitations (i.e., deficient uncertainties—see [37,38]) can have a null effect on trust or the perceived reliability of studies or findings [38]. For example, a recent experiment found no pattern of significant effects of deficient uncertainty [37]. This led to the following two alternative research hypotheses:

**Hypothesis** **1.1a.** *(Rational perspective): Reporting information on the limitations of included studies in the summary of a systematic review will have a positive main effect on the perceived tentativeness of the review findings.*

**Hypothesis** **1.1b.** *(Cognitive dissonance perspective): Reporting information on the limitations of included studies in the summary of a systematic review will have no main effect on readers’ perceived tentativeness of the review findings.*

These two contrasting perspectives also lead to different predictions about the effect of reporting information about limitations on the reader’s attitude toward the intervention, whose effectiveness is assessed in the systematic review. For illustrative purposes, suppose that while reading the summary of a systematic review on the efficacy of a vaccine, readers learn that the studies included in the review have several limitations. According to the rational perspective, this information should influence their attitude toward the vaccine because methodological limitations may bias the results demonstrating vaccine efficacy. The negative effect of portraying the deficient uncertainty of studies is feared by some science communication experts who do not wish to increase readers’ skepticism toward public policy targeting key issues such as climate change [39,40]. On the other hand, following the cognitive dissonance perspective, readers who strongly believe in the effectiveness of a policy intervention are likely to leave out that information to reduce the inconsistency between their belief and the content of the summary. The null effect hypothesis below (Hypothesis 1.2b) is supported by empirical evidence showing a tenuous effect of deficient uncertainty on behavioral intention [37].

**Hypothesis** **1.2a.** *(Rational perspective): Reporting information on the limitations of included studies in the summary of a systematic review will have a negative main effect on readers’ attitudes toward the intervention evaluated in the review.*

**Hypothesis** **1.2b.** *(Cognitive dissonance perspective): Reporting information on the limitations of included studies in the summary of a systematic review will have no main effect on readers’ attitudes toward the intervention evaluated in the review.*

While the methodological limitations of studies included in a systematic review refer to deficient uncertainty, the degree of agreement/disagreement in a body of evidence relates to consensus uncertainty [37,38]. In this experiment, we were interested in a situation where the results reported in a systematic review summary are characterized by a high degree of consensus demonstrating the effectiveness of a public health policy (vs. no evidence of such effectiveness, see Materials and Methods). A review of experiments on the effect of uncertainty found that consensus uncertainty is most clearly associated with negative main effects on various outcomes, such as trust, beliefs, and attitudes [38]. Furthermore, this review found no instance of consensus uncertainty resulting in positive main effects. An essential aspect of a systematic review of effectiveness is summarizing the findings regarding a given intervention’s effect on one or more outcome variables. Following the rational perspective (and based on existing empirical evidence in science communication), it is expected that the readers will process the information regarding the results about effectiveness, affecting their perceived tentativeness of review findings and their attitude toward the intervention.

**Hypothesis** **2.1.** *(Rational perspective): A systematic review summary that reports consensual findings demonstrating intervention effectiveness will decrease readers’ perceived tentativeness of review findings (compared to a summary with no evidence of effectiveness).*

**Hypothesis** **2.2.** *(Rational perspective): A systematic review summary that reports consensual findings demonstrating intervention effectiveness will lead to a more favorable attitude toward the intervention (than a summary with no evidence of effectiveness).*

In-depth case studies in the history of science show that well-established theoretical knowledge often outlasts contradictory empirical findings [41]. Furthermore, some researchers support interventions, although their results are not statistically significant [42]. The cognitive mechanism involved could be the propensity of individuals to reduce dissonance when confronted with counterintuitive information. Experimental-level findings show that exposure to contradictory or non-one-sided research findings lower both perceptions of information credibility and attitude toward the advocated behaviors [43]. Increased perceived efficacy of a specific vaccine was found among people who believe in vaccines efficacy in general, even after exposure to a blog reporting mixed efficacy and security findings [44]. These findings are consistent with the confirmation bias phenomenon that “once one has taken a position on an issue, one’s primary purpose becomes that of defending or justifying that position” [45]. Therefore, when a large consensus exists on the effectiveness of an intervention, a systematic review summary showing no evidence of intervention effectiveness could cause an increase in the perceived tentativeness of the review findings and a boost in attitude toward the intervention among readers knowledgeable on the subject; their expert knowledge will cause them to expect one-sided efficacy findings with no consensus uncertainty.

**Hypothesis** **3.1.** *(Cognitive dissonance perspective): A systematic review summary that shows no evidence of the effectiveness of an intervention—whose effectiveness is widely accepted in the literature (and among experts)—will positively affect the perceived tentativeness of the review findings among readers with reported expertise.*

**Hypothesis** **3.2.** *(Cognitive dissonance perspective): A systematic review summary that shows no evidence of the effectiveness of an intervention—whose effectiveness is widely accepted in the literature (and among experts)—will positively affect the attitude toward the intervention among readers with reported expertise.*

An experimental study on cognitive dissonance showed that people tend to search for consistency between their behavior, attitude, and beliefs [46]. This study found that eating meat reduced the perceived obligation to show moral concern for animals and cows’ moral status. In primary prevention related to nutrition, cross-sectional evidence shows that people with less healthy diets tend to be more skeptical about evidence-based nutrition messages, a phenomenon called nutrition backlash [47]. This phenomenon suggests that cognitive dissonance could arise when the individual’s behavior is incompatible with what is consistent with scientific evidence. When their behavior is related to the intervention assessed in the systematic review, one can expect that there will be a link between readers’ current behavior and their attitude toward the review findings and the intervention evaluated in the review. For example, suppose that the results reported in the review summary favor an intervention intended to promote the readers’ already adopted behavior. In this case, one can expect that such readers would see the review findings as less tentative (and favor the intervention), which will reinforce the coherence between their current behavior and the scientific information included in the summary. These considerations led to the following two alternative research hypotheses:

**Hypothesis** **4.1.** *(Cognitive dissonance perspective): Readers of a summary showing that an intervention effectively gets people to behave as they already do are less likely to perceive review findings as tentative than individuals whose behavior is inconsistent with the intervention.*

**Hypothesis** **4.2.** *(Cognitive dissonance perspective): Readers of a summary showing that an intervention is effective in getting people to behave as they already do are more likely to have a positive attitude toward the intervention evaluated in the review than individuals whose behavior is inconsistent with the intervention.*

## 2. Materials and Methods

### 2.1. Experimental Use Case: Bicycle Helmet Legislation

Road accidents involving bicycles occur regularly, sometimes resulting in severe head injuries or death [48,49]. As with seat belt legislation [50,51], some local, subnational, and national governments have introduced legislation for primary prevention purposes, mandating bicycle helmets for some or all cyclists [52]. A systematic review including several meta-analyses showed that helmet use is associated with reduced odds of head injury, including fatal ones [53]. For the current experiment, we selected a systematic review on the effectiveness of legislative interventions to increase bicycle helmet use among all age groups [54]. Since cycling is a general population activity, this choice allowed us to measure whether the respondent’s behavior was consistent with that promoted in the systematic review (i.e., wearing a helmet while cycling).

The chosen systematic review included eleven studies whose findings globally favor the legislation, as all studies show higher proportions of helmet use following legislation [54]. We designed four versions of the summary of this review using the template developed by [19] to meet the presentation needs expressed by health policy-makers. The systematic review used the number of people wearing helmets after the legislation as an outcome variable. All summary versions began with the same section labeled ‘Background’, starting with the following two sentences: “Head injuries related to cycling are frequent and can be serious. It is possible to prevent or reduce their severity by wearing a helmet”. On the survey’s last page, participants who read a manipulated version of the summary had access to the original version of the summary.

### 2.2. Participants and Recruitment

The criteria for selecting participants in this experiment were as follows: (1) individuals who self-identified as Americans or Canadians, (2) with access to a computer with Internet access, (3) who were not color-blind (since the summary contained colors), (4) 18 years of age or older, and (5) who reported having attained at least a bachelor’s degree. No participants reported being color-blind. We excluded participants who did not meet two specific information processing criteria: (1) devoting at least 70 s to the reading of the summary page, and (2) remaining within a 15-min time frame to complete the task. In total, the sample included 259 participants. We targeted university-educated individuals, since the content of a systematic review summary is more complex than that of a news article.

We used the Qualtrics paid opt-in online survey system for data recruitment and collection. This system randomly selects participants from traditional market research panels through email registration, web banners, social media, and invitation-only methods. To opt-in to a Qualtrics panel, respondents complete an online form including background information and agree to participate in online surveys conditional on an incentive. The potential participants were sent an invitation via email indicating that the survey was for research purposes only while informing them about its expected length and the incentive for participation. To avoid biases, the invitation email sent by Qualtrics to panelists did not include information about the topic of the experiment (i.e., bicycle helmet legislation). The sample was non-probabilistic and based on volunteering. However, Qualtrics randomly samples from a pool of potential respondents.

### 2.3. Study Design

We conducted a web-based randomized controlled experiment. The experiment had a 2 × 2 factorial design. We experimentally manipulated the amount of information on limitations (limitations disclosed vs. not disclosed) and the findings of studies included (findings favoring vs. not favoring legislation) in the systematic review on the effectiveness of bicycle helmet legislation in making cyclists wear a helmet [54]. Participants were randomly assigned to read one of the following four versions of the summary of the systematic review (i.e., see Figure S1 in Supplementary Materials):

- Version 1: Limitations + findings favoring helmet legislation
- Version 2: Limitations + findings not favoring helmet legislation
- Version 3: Limitations not revealed + findings favoring helmet legislation
- Version 4: Limitations not revealed + findings not favoring helmet legislation

We coded the experimental factor limitations 1 for participants allocated to version 1 or 2 of the summary and 0 for respondents assigned to version 3 or 4. We coded the experimental factor positive findings 1 for participants allocated to version 1 or 3 and 0 for those assigned to version 2 or 4.

We inserted limitations in summary versions 1 and 2 between the ‘Background’ and the ‘key messages’ sections. The participants could read the following paragraph accompanied by a logo calling attention to the limitations:

The absence of a control group was noted for several of the studies. While this absence is more problematic for studies of non-equivalent control groups, it can also be problematic for pre- and post-intervention studies (time bias). The analytical method used in some studies tends to mask temporary effects that occur immediately after the legislation is passed. It cannot be excluded that the absence of negative results is a consequence of a recognized tendency not to publish results showing no or negative effects. Finally, the failure to take into account certain factors could have biased the estimation of the effect of the legislation in the studies reviewed.

Both summary pages were displayed on the same page of the online questionnaire so that participants did not have to click on the “next” button to access the second page.

### 2.4. Procedure

Upon entering the online questionnaire administered under Qualtrics, participants were first exposed to an implied consent form, then asked for their highest level of education attained and whether they were color blind. Next, we instructed participants to carefully read the entire document to appear on the next page and then answer questions once they had finished reading. We warned participants to take their time to read the whole document, as it would not be possible to go back to the document afterward.

Qualtrics’s block randomizer for equal group size assigned each participant to one of the four conditions. Participants were not aware that they had been allocated to a group, thus ensuring allocation concealment. Each participant viewed only one version of the document. Participants’ blindness to experimental manipulation was given because the summary presented to them was not published.

Moreover, the original article published in *Injury Prevention* was not available on open access. It is, therefore, improbable that participants would have taken the time to compare the summary with the original article. The researchers had no control over the automated participant allocation process.

After answering the questions to measure the outcome variables, images representing each part of the summary were presented to participants in order of appearance in the summary. We asked participants to indicate (in order of perceived helpfulness) the three parts of the summary that they found most helpful in answering the previous questions (i.e., used to measure the outcome variables).

### 2.5. Measures

After viewing their assigned document, the participants answered all survey questions (presented in Supplementary Materials). The participants first answered the questions measuring the outcome variables. The perceived tentativeness of review findings was derived from a questionnaire [29] consisting of six different items (including three items with reverse wording) measured on a 7-point Likert scale, ranging from strongly disagree to strongly agree. The results of the principal component analysis indicate that the items measured two different constructs. Non-reverse items were strongly associated with the first dimension.

In contrast, reverse wording items were strongly associated with the second dimension, indicating a substantial likelihood of respondents’ confusion due to the reverse wording of some items. Thus, we constructed the perceived tentativeness index from the three items with no reverse wording (Cronbach’s alpha = 0.82). The perceived tentativeness was measured by taking the mean score of the following three items: (i) “The findings reported in the document are not really definitive”; (ii) “Based on this document, our understanding of bicycle helmet legislation is incomplete”; and (iii) “The findings reported in the document should only be considered preliminary”. We constructed the variable measuring the participants’ attitude toward the helmet legislation by taking the mean score of non-reverse wording items. These items were (i) “a bicycle helmet legislation is promising” and (ii) “a bicycle helmet legislation is certainly helpful” (Cronbach’s Alpha = 0.86). We took these items from [29]’s instrument.

We measured self-reported expertise in helmet legislation by asking participants whether this type of intervention falls within their field of professional expertise. To test the fourth set of hypotheses, we first asked participants how often they had cycled in the past 12 months. Next, we asked those who indicated they had cycled at least once how often they wore a helmet. From the responses to these two questions, a nominal variable was created with the following mutually exclusive categories: (i) never rode a bike; (ii) never wore a helmet while riding a bike; (iii) wore a helmet sporadically; and (iv) always wore a helmet.

### 2.6. Data Treatment and Analysis

We processed and analyzed the data using RStudio. We used R and Latex programming to perform all operations on the dataset. We performed descriptive analyses of the sample, randomization checks, and linear regression analyses (some with interaction terms) to test research hypotheses. We produced all analysis tables with the *R Stargazer* [55] and Reporttools [56] packages.

## 3. Results

### 3.1. Sample Characteristics

Table 1 presents the sample characteristics. The sample was 51.4% female, and the largest age group was 35–54 years old (39.4%), while the smallest was 75 years old and over (4.2%). Regarding education level, 38.2% had completed a master’s or doctorate. Table 2 shows the descriptive statistics for the outcome variables.

The number of participants in each group was almost equal (a slight unbalance was due to applying the exclusion criteria). The two groups assigned to the summary with limitations (and positive or non-significant findings) were of equal size (64 participants; 24.7% each). The group allocated to the summary without limitations and positive intervention findings had 62 participants (23.9%). The group assigned to the summary without limitations and non-significant intervention findings included 69 participants (26.6%). Half of the sample was exposed to a summary version with limitations (49.4%). Almost the same distribution was observed for the portion of the sample exposed to a summary version reporting significant positive effects of helmet legislation (48.6%). Overall, the groups were similar with respect to the exposure to experimental manipulations.

As for the variables measured by observation to test the third and fourth sets of hypotheses, the sample included 16.6% of participants with reported expertise in helmet legislation. About one-third of the sample said they had not ridden a bicycle in the past 12 months (34.0%), while 15.1% never wore a helmet, 20.9% wore it sporadically, and 30.1% said they always wore it.

### 3.2. Regression Results: Hypothesis Testing

Table 3 reports the results of the additive linear regression models for the first two sets of research hypotheses. These hypotheses predicted a main effect, and alternatively, the absence of the main effect of the experimental factors on perceived tentativeness and attitude toward the intervention. We explored interaction effects between the two experimental factors and found no effects (interaction effect on perceived tentativeness: −0.116, CI 95% from −0.780 to 0.547; on attitude: −0.230, CI 95% from −0.901 to 0.442). The results presented in Table 3 are those of the model without interaction effects. They show that exposure to a summary including information on the limitations had neither an effect on perceived tentativeness (0.218; CI 95% from −0.113 to 0.549) nor on attitude toward helmet legislation (0.236; CI 95% from −0.099 to 0.572).

The regression coefficient linking the variable positive findings to perceived tentativeness was not significant (−0.280; CI 95% from −0.611 to 0.051), whereas the coefficient relating to attitude was significant and positive (0.788; CI 95% from 0.453 to 1.123). The experimental factors explained almost none of the variance in perceived tentativeness, as illustrated by an adjusted R-squared of about 1%. In contrast, alone they explained about 8% of the variance in attitude toward helmet legislation.

The last two sets of research hypotheses provided additional opportunities to test the alternative theoretical perspective of a rational readership vs. a readership influenced by cognitive dissonance. Table 4 and Table 5 present the results of this hypothesis testing.

We found mixed results for the third set of hypotheses. The two hypotheses in this set implied an interaction effect involving exposure to favoring findings and self-reported expertise. The results presented in Table 4 show no interaction effect on perceived tentativeness (0.091; CI 95% from −0.771 to 0.954). However, reported expertise had a main effect on perceived tentativeness. We re-estimated the regression model without the interaction term, since the coefficient of self-reported expertise could not be interpreted as an average main effect when included with the interaction term. This re-estimation showed that self-reported expertise in helmet legislation had a positive and significant main effect on the perceived tentativeness (0.990; CI 95% from 0.530 to 1.450).

Furthermore, the results presented in Table 4 show an interaction effect involving self-reported expertise and the manipulation of the direction of the review findings on attitude toward helmet legislation (−1.429; CI 95% from −2.264 to −0.595). The negative sign of the interaction coefficient indicates that self-reported expertise reduced the positive effect that exposition to ‘findings favoring legislation’ had on attitude toward helmet legislation. In addition, the main effect coefficient for the variable ‘self-reported expertise’ was positive and significant (1.964; 95% CI from 1.368 to 2.560; this coefficient is not the average main effect of self-reported expertise due to the inclusion of the interaction effect parameter in the linear regression model). One can interpret this coefficient as follows: self-reported expertise positively affected attitude toward helmet legislation among participants exposed to the summary showing a non-significant effect of mandatory helmet legislation on helmet use. The ‘positive findings’ variable forms an interaction effect with the ‘self-reported expertise’ variable. Therefore, its main effect coefficient (1.092; 95% CI from 0.755 to 1.429) means that exposure to the summary presenting a positive effect of bicycle helmet legislation on helmet use positively affects attitude toward helmet legislation among participants who did not report having expertise on this intervention.

The results of the fourth and final set of research hypotheses were not as expected. As shown in Table 5, the interaction between helmet use and favoring findings did not affect either perceived tentativeness or attitude. This expectation was in line with Hypotheses 4.1 and 4.2. Readers assigned to a summary with positive findings who cycled with a helmet would perceive the review findings as less tentative and have a more favorable attitude toward such legislation than readers who cycle without wearing a helmet. Interestingly, the results suggest that helmet use had an average main effect on attitude toward helmet legislation, regardless of the summary version presented to the participants. We, therefore, re-estimated the regression equations without specifying any interaction between helmet use and positive findings (the table of results is not presented for space reasons).

The results show that the average main effect of helmet use on perceived tentativeness was not significant. Wearing the helmet sporadically (−0.218; 95% CI from −0.796 to 0.359) or always (0.272; 95% CI from −0.260 to 0.805) rather than never (the reference) was not significantly associated with perceived tentativeness. However, we found that always wearing a helmet while cycling increased the attitude toward helmet legislation by an average of 1 point on a scale of 1 to 7 (1.004; 95% CI from 0.485 to 1.524). Although lower, sporadically wearing a helmet also increased the attitude toward helmet legislation by a statistically significant amount (0.591; 95% CI from 0.028 to 1.154). Never having ridden a bicycle in the last 12 months rather than having ridden without a helmet was not associated with the attitude toward helmet legislation (−0.025; 95% CI from −0.541 to 0.490).

### 3.3. Exploring Potential Mechanisms

Table 6 presents the frequency distributions of the binary variable capturing the section of the summary of the systematic review that was considered most helpful by the participants exposed and not exposed to the paragraph on limitations (Section 2 of the summary). For participants exposed to a summary with the limitations section, this section was far from being the one that was considered the most helpful by respondents in answering the questions measuring the outcome variables. Specifically, only 15 of the 128 participants (11.7%) in a trial group with a summary version including limitations rated Section 2 as the most helpful for answering the questions.

Among participants exposed to information on limitations, the section they most often perceived as the most helpful was the one presenting the key messages from the systematic review (45/128; 35.2%), followed by the first section of the summary (background section) (31/128; 24.2%) and the section presenting the results of the systematic review in bullet points (19/128; 14.8%). The table showing the statistical results of the included studies was considered the most helpful section of the summary by only 6 of the 128 participants (4.7%) exposed to information on limitations. The remaining sections (much shorter and partly located at the end of the summary) were considered the most helpful by very few or none of the participants.

For participants exposed to a summary that did not include the limitations section, the three areas most often considered the most helpful were the same as those exposed to Section 2 of the summary. In effect, the section most often perceived as the most helpful was the one presenting the key messages from the systematic review (46/131; 35.1%), followed by the first section of the summary (background section) (33/131; 25.2%) and the section presenting the results of the systematic review in bullet points (27/131; 20.6%). The image representing a thumb up accompanied by a short positive sentence was the summary area that was considered the most helpful by 17 of the 131 participants in a trial group with no exposure to information on limitations (13%). The thumb down section presented to the right of the thumb up area was considered the most helpful by only two participants (1.5%). Participants exposed to the limitations were half as likely (6.2%) to feel that the thumb up section was the most helpful area. Regarding the thumb down section, almost the same proportion was observed (1.6% vs. 1.7%).

## 4. Discussion

This experiment suggests that including textual information on the methodological limitations of studies in the summary of a systematic review about legislation on bicycle helmets had no effect on the perception of the tentativeness of the review findings nor on attitude toward the legislation. This finding departs from the view of rational readership that should ideally process and consider deficient uncertainty. However, this null effect finding for manipulating deficient uncertainty has also been found in published science communication experiments, along with positive and negative effects [38]. The mechanisms that explain these mixed findings for the communication of deficient uncertainty deserve to be explored in meta-research studies. Such studies would allow for examining the experiments’ characteristics associated with these mixed effects. In our experiment, a possible explanation of the null effect that exposure to deficient uncertainty had on the perceived tentativeness and the attitude toward the legislation might be that, although presented at the beginning of the summary, the paragraph on the limitations of the studies did not seem to catch the attention of most readers. The sections before (i.e., background information about helmet use and legislation) and right after (i.e., key messages, essentially the main results, not the limitations) were primarily considered the ones with more helpful information.

There may be numerous reasons why such informational contents were not deemed as useful by participants: the nature of the text, its inherent level of complexity, specific lexical, semantic, and syntactic features [57,58], the display format (i.e., font size, form and type, presence of colored design elements and pictures), or individual differences in the ability of participants to comprehend reading material (e.g., see [59]). It should also be noted that the types of effects examined in this study have been almost exclusively tested using news articles. To our knowledge, the current experiment is the first to have tested them using the summary of a systematic review. News articles are more accessible and contain less technical jargon than the summary presented to participants in our study, which may also result in many participants not considering the information on limitations. Examining the readability and linguistic characteristics of different textual summary formats of Cochrane systematic reviews, it was found that all formats had low readability, and that sadness was the most frequent perceived emotional tone [59]. This study also found that the press release format was perceived as more engaging than the scientific and plain language summaries. One of the practical implications of these results might be to professionalize the writing of systematic review summaries to increase their readability and induce pleasant emotions in readers.

Another possibility is that, unlike the description of the main results, information on methodological limitations is not directly related to the primary purpose of the systematic review, which is to determine the effectiveness of an intervention. Information on methodological shortcomings may thus be considered less relevant than effectiveness findings. The question of the relative importance of different parts of the content of a systematic review summary will require future study.

Similar to an experiment that examined the same set of dependent variables as [29], no interaction effect between deficient and consensus uncertainty was found in this study. This suggests that in a written scientific communication context, deficient uncertainty does not moderate the effect that consensus uncertainty has on the perception of tentativeness of communicated findings and attitudes towards a policy intervention.

The study also shows that displaying the results in such a way that they show a broad consensus in favor of the effectiveness of the intervention has a positive effect on people’s attitudes toward the intervention. This finding is consistent with experiments showing that consensus uncertainty backlashes toward the promoted intervention [60,61]. In this study, participants exposed to a summary portraying the efficacy findings as non-significant had, on average, a more negative attitude toward helmet legislation. On the other hand, interaction effect analysis showed that among those with reported professional expertise on helmet legislation, manipulation of review findings to show insignificant findings did not appear to diminish their approval of the intervention. Cognitive dissonance and the belief disconfirmation phenomenon can sometimes promote rational information processing by allowing individuals to maintain their prior beliefs in the face of contradictory misinformation. However, further research would be necessary to assess how cognitive strategies to maintain prior beliefs impact scientific information processing (e.g., see [62]). It should be noted that self-reported expertise was the only significant predictor of the perception of the tentativeness of the review findings, which suggests that prior knowledge stimulates a critical attitude toward scientific studies (i.e., bearing in mind that the reported expertise is a surrogate of the actual objectively measured expertise).

Another finding provides support for the theoretical perspective of cognitive dissonance. Participants who wear helmets while cycling had a more favorable attitude toward helmet legislation than those who cycle without a helmet. This conclusion applied to the whole set of participants, regardless of their assigned summary version. This finding may be due to the inconsistency or coherence between the readers’ behavior and the one promoted by the intervention under evaluation in the review. People who already behave in the promoted way are more likely to favor the intervention. This phenomenon is well-known in advertising and seems to apply to science communication. On a practical level, based on segmentation analyses, science communicators may wish to consider tailoring their content based on the known behavior of different audiences.

A strength of this study is the use of a systematic review summary to test hypotheses deduced from established cognitive science theories and grounded in recent empirical studies in science communication. There is a rich niche of experimental studies focusing on science communication through media articles and blogs. Developers and experimenters of systematic review summaries might benefit from increased dialogue with this research community.

One limitation of this study is the lack of control over the environment where participants performed the experimental tasks. This issue applies for any web-based experiment where the environment in which participants fill in questionnaires is unknown. Furthermore, except for the experimental manipulations, this type of experiment only collects self-reported data. In that respect, objective measures of behavior (such as eye-movement tracking or online physiological markers of participant’s functional state) would reduce interference in measuring causal links between experimental manipulations and outcomes. Moreover, restricting the study population to individuals with a university education was necessary because a systematic review summary is more technical than a news article. However, scientific communication cannot be limited to this population, and future studies should target more specific populations such as decision-makers. Conducting experiments within the governmental apparatus is feasible (and has already been done), but it requires going through many administrative steps that can sometimes be cumbersome and tedious for researchers. Since many more experiments are needed to increase understanding of the phenomena examined in this study, a promising avenue would be to recruit government agents by purchasing access to panels from survey companies. Such a protocol would significantly speed up the data collection process and, consequently, the accumulation of knowledge.

## 5. Conclusions

The information contained in a systematic review summary did not have a consistent impact on readers. Additional research is required to better understand the influence of scientific information on people’s perception of preventive public health interventions.

## Figures and Tables

**Table 1:** Sample characteristics.

Variable	Levels	*N*	%
Limitations	No limitation reported	131	50.6
	Limitations reported	128	49.4
	All	259	100
Findings	Non-significant	133	51.4
	Positive	126	48.6
	All	259	100
Expertise	No expertise reported	216	83.4
	Self-reported expertise	43	16.6
	All	259	100
Helmet	Never used the bike	88	34.0
	Never wore a helmet	39	15.1
	Wore the helmet sporadically	54	20.9
	Has always worn the helmet	78	30.1
	All	259	100
Education	Bachelor	160	61.8
	Master or PhD	99	38.2
	All	259	100
Sex	Male	125	48.6
	Female	132	51.4
	All	257	100
Age	18–34 years old	72	27.8
	35–54 years old	102	39.4
	55–74 years old	74	28.6
	75+ years old	11	4.2
	All	259	100

**Table 2:** Descriptive statistics for outcome variables.

Variable	*N*	Min	Max	Mean	Standard Deviation	Missing Values
Perceived Tentativeness of Review Findings	259	1	7	4.7	1.4	0
Attitude Toward Helmet Legislation	259	1	7	5.1	1.4	0

**Table 3:** OLS regression results for first and second sets of research hypotheses involving experimental factors.

	Dependent Variable:	
	Perceived Tentativeness of Review Findings	Attitude toward Bicycle Helmet Legislation
	(1)	(2)
	Coefficient (95% Confidence Interval)	Coefficient (95% Confidence Interval)
Reported limitations	0.218 (−0.113, 0.549)	0.236 (−0.099, 0.572)
Positive findings	−0.280 (−0.611, 0.051)	0.788 ** (0.453, 1.123)
Constant	4.679 ** (4.399, 4.960)	4.612 ** (4.328, 4.896)
Observations	259	259
R^2^	0.017	0.084
Adjusted R^2^	0.009	0.077

Note: * *p* < 0.05; ** *p* < 0.01.

**Table 4:** OLS regression results for the third set of research hypotheses involving self-reported expertise in helmet legislation.

	Dependent Variable:	
	Perceived Tentativeness of Review Findings	Attitude toward Bicycle Helmet Legislation
	(1)	(2)
	Coefficient (95% Confidence Interval)	Coefficient (95% Confidence Interval)
Positive findings	−0.261 (−0.609, 0.087)	1.092 ** (0.755, 1.429)
Self-reported expertise	0.947 ** (0.331, 1.563)	1.964 ** (1.368, 2.560)
Master or PhD	−0.163 (−0.509, 0.182)	0.013 (−0.321, 0.347)
Female	−0.322 (−0.655, 0.011)	0.028 (−0.295, 0.350)
35–54 years old	−0.067 (−0.459, 0.324)	−0.401* (−0.780, −0.022)
55–74 years old	−0.243 (−0.676, 0.190)	−0.161 (−0.580, 0.258)
75+ years old	−0.179 (−1.040, 0.682)	−0.390 (−1.224, 0.444)
Positive findings * Self-reported expertise	0.091 (−0.771, 0.954)	−1.429 ** (−2.264, −0.595)
Constant	4.954 ** (4.493, 5.415)	4.579 ** (4.133, 5.025)
Observations	257	257
R2	0.112	0.246
Adjusted R^2^	0.083	0.222

Note: * *p* < 0.05; ** *p* < 0.01.

**Table 5:** OLS regression results for the fourth set of research hypotheses.

	Dependent Variable:	
	Perceived Tentativeness of Review Findings	Attitude toward Bicycle Helmet Legislation
	(1)	(2)
	Coefficient (95% Confidence Interval)	Coefficient (95% Confidence Interval)
Positive findings	−0.324 (−1.187, 0.538)	0.835 * (0.007, 1.663)
Never rode a bike	0.154 (−0.517, 0.826)	−0.313 (−0.957, 0.332)
Wore the helmet sporadically	0.005 (−0.825, 0.835)	1.102 ** (0.305, 1.899)
Always wore the helmet	0.088 (−0.601, 0.777)	1.181 ** (0.520, 1.843)
Master or PhD	−0.037 (−0.399, 0.325)	0.019 (−0.328, 0.367)
Female	−0.457 * (−0.806, −0.108)	0.011 (−0.324, 0.346)
35–54 years old	−0.123 (−0.534, 0.288)	−0.240 (−0.634, 0.155)
55–74 years old	−0.507 * (−0.998, −0.017)	0.074 (−0.397, 0.545)
75+ years old	−0.461 (−1.387, 0.466)	−0.188 (−1.077, 0.701)
Positive findings * Never rode a bike	0.011 (−1.015, 1.038)	0.602 (−0.384, 1.587)
Positive findings * Wore the helmet sporadically	−0.311 (−1.450, 0.828)	−0.767 (−1.860, 0.327)
Positive findings * Always wore the helmet	0.420 (−0.632, 1.471)	−0.343 (−1.352, 0.667)
Constant	5.177 ** (4.509, 5.846)	4.368 ** (3.727, 5.010)
Observations	257	257
R2	0.074	0.228
Adjusted R^2^	0.029	0.190

Note: * *p* < 0.05; ** *p* < 0.01.

**Table 6:** Most helpful summary section for summary including information about limitations.

	Summary with a Section on Limitations					
	Yes			No		
Summary Section	*n*	%	Sum%	*N*	%	Sum%
Background	31	24.2	24.2	33	25.2	25.2
Limitations of included studies	15	11.7	35.9	NA	NA	NA
Key messages	45	35.2	71.1	46	35.1	60.3
Thumb up	8	6.2	77.3	17	13.0	73.3
Thumb down	2	1.6	78.9	2	1.5	74.8
Results in bullets	19	14.8	93.8	27	20.6	95.4
Results in table	6	4.7	98.4	4	3.0	98.5
Review method	0	0.0	98.4	1	0.8	99.2
Review funding	2	1.6	100	1	0.8	100
Summary authors	0	0.0	100	0	0.0	100

## Data Availability

The dataset used during the current study is available from the senior author upon reasonable request.

## Supplementary Materials

The following supporting information can be downloaded at: https://www.mdpi.com/article/10.3390/ijerph19106234/s1, Figure S1: Summary with information on limitations and positive findings; Table S1: Survey questions; Table S2: Randomization check for the factor measuring exposition vs. non-exposition to information about limitations; Table S3: Randomization check for the factor measuring exposition vs. non-exposition to positive review findings.
