# Supplementary material for: The Impact of Information Presentation and Cognitive Dissonance on Processing Systematic Review Summaries: A Randomized Controlled Trial on Bicycle Helmet Legislation
Source: Int J Environ Res Public Health. 2022 May 20;19(10):6234. doi: 10.3390/ijerph19106234 (PMC9140747; doi:10.3390/ijerph19106234)
Supplement: Supplementary file 1 [file ijerph-19-06234-s001.zip › ijerph-1707330-supplementary.pdf]

Supplementary Material

Effectiveness of bicycle helmet legislation to increase helmet use: a systematic review

2019

Reference -> Karkhaneh, M., Kalenga, J. C., Hagel, B. E., & Rowe, B. H. (2006). Effectiveness of bicycle helmet legislation to increase helmet use: a systematic review. Injury Prevention, 12(2), 76-82.

Background: Head injuries related to cycling are frequent and can be serious. It is possible to prevent or reduce their severity by wearing a helmet. However, the use of bicycle helmets is infrequent in most developed countries. Therefore, the adoption of helmet legislation is presented as a way to increase helmet use. Although this intervention is considered effective, no systematic review of the scientific literature was conducted until 2006. The systematic review summarized in this document assesses the research evidence related to the effectiveness of this type of legislation on helmet use in several age groups.

Risk of bias in the studies reviewed: The absence of a control group was noted for several of the studies. While this absence is more problematic for studies of non-equivalent control groups, it can also be problematic for pre- and post-intervention studies (time bias). The analytical method used in some studies tends to mask temporary effects that occur immediately after the legislation is passed. It cannot be excluded that the absence of negative results is a consequence of a recognized tendency not to publish results showing no or negative effects. Finally, the failure to take into account certain factors could have biased the estimation of the effect of the legislation in the studies reviewed.

Key messages for public policy

These are the first efforts to produce a systematic review of the effectiveness of bicycle helmet legislation in increasing helmet use. The main results of this systematic review are as follows:

- All twelve studies identified in the systematic review conclude that the adoption of such legislation increases helmet use in the community (municipal, regional or provincial). These results are based on impact measures collected in the short and long term. In the long term (12 months and more), the effects of the legislation were maintained.
- While the results suggest that the adoption of helmet legislation increases helmet use in the population, the magnitude of the effects measured varies significantly across studies (between +5 and +54).

Potential applications:

- This type of legislation seems to increase the use of helmets. Subgroup analysis suggests that the effect of the legislation may be lower when basic helmet use rates are already high in the population. However, this does not mean that the intervention should be abandoned in these settings.

Additional Interpretations:

- The impact of the legislation appears to be lower in regions with a higher basic helmet use rate and in regions with a high socio-economic status.
- There is little evidence on the effect of legislation targeting helmet use in children on helmet use in adults. A study shows that there is no effect.

The results of the systematic review suggest that the main effect of adopting a helmet legislation may be the awareness and education that such a law provides in the population and among parents on the benefits of helmet use.

The effect of the legislation appears to be weaker in regions where helmet use is initially most common and in regions with high socio-economic status.

Results

- Before the law was passed, the rate of helmet use ranged from 4% to 59% and, after the law was passed, it ranged from 37% to 91%, depending on the studies.
- In practice, the use of helmets is four times more important after than before the adoption of the legislation.
- Nine out of 12 studies show an increase in the long-term effects (beyond one year) of the legislation compared to the short-term effects (one year or less after the adoption of the legislation).
- In one study, the adoption of helmet legislation only had an impact on children (persons under 18 years of age).
- Four out of twelve studies examined the data by gender. These analyses did not identify any such influence on the relationship between legislation and helmet use.
- Higher basic helmet wearing rates may be associated with lower effectiveness of the adoption of helmet legislation.

Table 1. - Changes in helmet wearing rates before and after legislation in the 12 studies reviewed

Publication reviewed	Period covered by the measurement of post-legislation effects	Proportion before legislation (%)	Proportion after legislation (%)	Δ
Cole (1992)	Less than a year	4	47	+ 43
Cameron (1994)	Two years	31	75	+ 44
Ni (1997)	One year	24	51	+ 27
Foss (2000)	Four years	39	60	+ 21
Kanny (2001)	Two years	33	79	+ 46
Leblanc (2002)	Two years	38	84	+ 46
Thomas (2002)	Less than a year	32	37	+ 5
Delamaster (2003)	Four years	7	61	+ 54
Liller (2003)	-*	7	57	+ 50
Parkin (2003)	Two years	44	66	+ 22
Hagel (2005)	Two years	43	53	+ 10
Povey (2005)	Eleven years	59	91	+ 32

* The population of this study was not available and was therefore excluded from the analysis.

Methodology used to produce the systematic literature review

Based on research in ten bibliographic databases and grey literature, 12 studies were selected. The publications included are cohort studies, before and after with a control group, interrupted time series studies or non-equivalent control group studies that examine the effect of introducing legislation on bicycle helmet use at the regional, provincial or municipal level. The quality assessment of the 12 studies was conducted using a modified version of the Downs and Black checklist. The statistical analysis focused on changes in the proportions of helmet use among pre- and post-legislation cyclists. Odds ratios were calculated based on the demographic data available for the intervention and control groups or pre- and post-legislative studies.

Funding of the systematic review

The systematic review was funded by the Canadian Institutes of Health Research (Ottawa, ON) and the Children's Hospital Foundation (Calgary, AB).

This summary was produced by the Centre d'analyse des politiques publiques de l'Université Laval

UNIVERSITÉ LAVAL CAPP Centre d'analyse des politiques publiques

Figure S1. Summary with information on limitations and positive intervention findings. The four versions of the summary were two page long, but participants simply had to scroll down the page to view the second page. Both pages of the summary were presented on the same page on the survey platform.

Table S1. Survey questions

Question	***Purpose of the question***
What is the highest you have achieved?<br>• High school (Not eligible to answer the questionnaire)<br>• Bachelors<br>• Masters<br>• PhD	Eligibility & control variable 'Education level'. Also used to perform randomization check.
Are you color-blind? In other words, do you have difficulty seeing colors?<br>• Yes [Not eligible to answer the questionnaire]<br>• No	Eligibility
Please indicate to what extent you agree with the following statements:<br>• The findings reported in the document are not really definitive<br>• Based on this document, our understanding of bicycle helmet legislation is incomplete<br>• The document is conclusive [reversed item]<br>• The findings reported in the document are reliable [reversed item]<br>• The document provides a strong basis for deciding whether or not to adopt bicycle helmet legislation in the future [reversed item]<br>• The findings reported in the document should only be considered preliminary	Outcome variable: Perceived tentativeness
Please indicate to what extent you agree with the following statements:<br>• A bicycle helmet legislation is promising<br>• A bicycle helmet legislation is risky [reversed item]<br>• A bicycle helmet legislation is certainly helpful<br>• The risks related to bicycle helmet legislation are greater than the benefits [reversed item]<br>• If a loved one had a need for which bicycle helmet legislation is one of the solutions, I would like him or her to benefit from it<br>• If I had a need for which this bicycle helmet legislation is one of the solutions, I would like to benefit from it<br>• I find concerning the idea of implementing bicycle helmet legislation [reversed item]	Outcome variable: Attitude toward helmet legislation
Below you will find the document you have read divided into ten parts. ***Please rank the three parts*** *from* ***1*** *(****the most helpful*** *to answer the questions you have just answered after reading the document) to* ***3*** *(****the third most helpful*** *to answer the questions you have just answered after reading the document).*	*Explore which part of the summary captured the most attention*

Over the last 12 months, about how often did you ride a bicycle?<br>• Daily (multiple times a day, most days in the last 12 months)<br>• Weekly (multiple times a week, most weeks in the last 12 months)<br>• Monthly (from time to time, most months in the last 12 months)<br>• Quarterly (a few times during the last 12 months)<br>• Yearly (only once during the last 12 months)<br>• Never	Question used to create the helmet use variable to test the fourth set of hypotheses
Over the last 12 months, how often did you wear a helmet when riding a bicycle?<br>• Always wore a helmet<br>• More than half of the time<br>• About half of the time<br>• Less than half of the time<br>• Never wore a helmet	Question used to create the helmet use variable to test the fourth set of hypotheses
Is bicycle helmet legislation in your field of professional expertise?<br>• Yes<br>• No	Question used to measure the variable self-reported expertise to test the third set of hypotheses
What sex were you assigned at birth?<br>• Male<br>• Female<br>• Intersex<br>• Pref not to answer	Control variable entered in regression models for testing the third and fourth sets of hypotheses. Also used to perform randomization check.
Which age group do you fall into?<br>• Less than 18 years old<br>• Between 18 and 24 years old<br>• Between 25 and 34 years old<br>• Between 35 and 44 years old<br>• Between 45 and 54 years old<br>• Between 55 and 64 years old<br>• Between 65 and 74 years old<br>• Between 75 and 84 years old<br>• 85+ years old	Control variable entered in regression models for testing the third and fourth sets of hypotheses. Also used to perform randomization check.

Table S2. Randomization check for the factor measuring exposition vs. non-exposition to information about limitations

Variable	Levels	n Summary without the section on limitations	% Summary without the section on limitations	n Summary with the section on limitations	% Summary with the section on limitations	n All	% all
Expertise	No	109	83.2	107	83.6	216	83.4
	Yes	22	16.8	21	16.4	43	16.6
p = 1.00	all	131	100	128	100	259	100
Helmet use	Never used a bike	50	38.2	38	29.7	88	34.0
	Never wore a helmet	21	16.0	18	14.1	39	15.1
	Wore it sporadically	24	18.3	30	23.4	54	20.9
	Has always worn it	36	27.5	42	32.8	78	30.1
p = 0.40	all	131	100	128	100	259	100
Education	Bachelor	80	61.1	80	62.5	160	61.8
	Master or PhD	51	38.9	48	37.5	99	38.2
p = 0.91	all	131	100	128	100	259	100
Sex	Male	64	49.6	61	47.7	125	48.6
	Female	65	50.4	67	52.3	132	51.4
p = 0.85	all	129	100	128	100	257	100
Age	18-34	33	25.2	39	30.5	72	27.8
	35-54	49	37.4	53	41.4	102	39.4
	55-74	44	33.6	30	23.4	74	28.6
	75+	5	3.8	6	4.7	11	4.2
p = 0.34	all	131	100	128	100	259	100

Table S3. Randomization check for the factor measuring exposition vs. non-exposition to positive intervention findings

Variable	Levels	n Summary with non-significant findings	% Summary with non-significant findings	n Summary with positive findings	% Summary with positive findings	n All	% all
Expertise	No	109	82.0	107	84.9	216	83.4
	Yes	24	18.0	19	15.1	43	16.6
p = 0.64	all	133	100	126	100	259	100
Helmet use	Never used a bike	48	36.1	40	31.8	88	34.0
	Never wore a helmet	24	18.1	15	11.9	39	15.1
	Wore it sporadically	19	14.3	35	27.8	54	20.9
	Has always worn it	42	31.6	36	28.6	78	30.1
p = 0.05	all	133	100	126	100	259	100
Education	Bachelor	82	61.6	78	61.9	160	61.8
	Master or PhD	51	38.4	48	38.1	99	38.2
p = 1.00	all	133	100	126	100	259	100
Sex	Male	61	46.2	64	51.2	125	48.6
	Female	71	53.8	61	48.8	132	51.4
p = 0.50	all	132	100	125	100	257	100
Age	18-34	32	24.1	40	31.8	72	27.8
	35-54	52	39.1	50	39.7	102	39.4
	55-74	44	33.1	30	23.8	74	28.6
	75+	5	3.8	6	4.8	11	4.2
p = 0.34	all	133	100	126	100	259	100
